# Fatty acids from fish or vegetable oils promote the adipogenic fate of mesenchymal stem cells derived from gilthead sea bream bone potentially through different pathways

**DOI:** 10.1371/journal.pone.0215926

**Published:** 2019-04-24

**Authors:** Natàlia Riera-Heredia, Esmail Lutfi, Joaquim Gutiérrez, Isabel Navarro, Encarnación Capilla

**Affiliations:** Department of Cell Biology, Physiology and Immunology, Faculty of Biology, University of Barcelona, Barcelona, Spain; Universidade de Vigo, SPAIN

## Abstract

Fish are rich in n-3 long-chain polyunsaturated fatty acids (LC-PUFA), such as eicosapentaenoic (EPA) and docosahexaenoic (DHA) acids, thus they have a great nutritional value for human health. In this study, the adipogenic potential of fatty acids commonly found in fish oil (EPA and DHA) and vegetable oils (linoleic (LA) and alpha-linolenic (ALA) acids), was evaluated in bone-derived mesenchymal stem cells (MSCs) from gilthead sea bream. At a morphological level, cells adopted a round shape upon all treatments, losing their fibroblastic form and increasing lipid accumulation, especially in the presence of the n-6 PUFA, LA. The mRNA levels of the key transcription factor of osteogenesis, *runx2* significantly diminished and those of relevant osteogenic genes remained stable after incubation with all fatty acids, suggesting that the osteogenic process might be compromised. On the other hand, transcript levels of the main adipogenesis-inducer factor, *pparg* increased in response to EPA. Nevertheless, the specific PPARγ antagonist T0070907 appeared to suppress the effects being caused by EPA over adipogenesis. Moreover, LA, ALA and their combinations, significantly up-regulated the fatty acid transporter and binding protein, *fatp1* and *fabp11*, supporting the elevated lipid content found in the cells treated with those fatty acids. Overall, this study has demonstrated that fatty acids favor lipid storage in gilthead sea bream bone-derived MSCs inducing their fate into the adipogenic *versus* the osteogenic lineage. This process seems to be promoted via different pathways depending on the fatty acid source, being vegetable oils-derived fatty acids more prone to induce unhealthier metabolic phenotypes.

## Introduction

In the last decades, both the world population and the consumption of fish and seafood per capita have increased and will continue to rise. Fish products are rich in n-3 long chain polyunsaturated fatty acids (LC-PUFA) such as eicosapentaenoic (EPA, 20:5n-3) and docosahexaenoic (DHA, 22:6n-3) acids [1], which are crucial nutrients for overall health [2]. For these reasons, scientific research is indispensable to improve aquaculture production under sustainable conditions, which implies among others, a reduction in the use of fish oil in aquafeeds formulation [3]. The alternatives are vegetable oils, which in contrast to fish oil, are richer in n-6 or n-9 PUFA such as linoleic (LA, 18:2n-6), oleic (18:1n-9) or alpha-linolenic (ALA, 18:3n-3) acids [4]. Moreover, fish (especially marine) may have limited ability to convert C_18_ PUFA to C_20/22_ [4], [5] so, it should be considered that feeding fish with highly substituted diets can result in tissues with lower n-3 LC-PUFA content [6], [7]. Apart from changes in the fatty acid composition of the fish filet [8], [9], [10], dietary vegetable oils in excess can cause adipose tissue and hepatic metabolic alterations [11], [12] or affect the immune system [13], [14]. Besides, low concentrations of dietary EPA and DHA during development, have been related to increased incidence of skeletal malformations [15], [16]. Overall, these can lead to unhealthier or low-quality fish having consequences in aquaculture production.

Fish bone consists, as in other vertebrates, of several cell types including progenitor cells or mesenchymal stem cells (MSCs) that differentiate into osteoblasts after appropriate induction [17], [18]. There are many regulators involved in the process of osteoblastogenesis, but runt-related transcription factor 2 (Runx2), is the main transcription factor controlling lineage determination and osteogenic genes expression [19]. Once differentiated, osteoblasts produce the bone extracellular matrix (ECM) or osteoid, where key components such as osteonectin (ON), osteopontin (OP) and osteocalcin subsequently regulate mineral deposition [20], [21], [22]

Interestingly, mammalian adipocytes can arise from the same MSCs as osteoblasts and a high degree of plasticity has been observed between the two cell lineages, even in very advanced maturation stages [23]. During the onset of the adipogenic process, transcription factors such as CCAAT/enhancer binding protein β and δ (C/EBPβ and C/EBPδ) are activated, which in turn, induce the expression of *c/ebpa* and peroxisome proliferator-activated receptor γ (*pparg*) [24]. These factors successively promote the transcription of specific genes mainly related with lipid metabolism like fatty acid synthase (*fas*) or the hormone sensitive lipase (*hsl*) [25]. Adipose tissue can grow not only by elevating the cellular number from resident precursors (hyperplasia) but also by increasing the size of existing adipocytes (hypertrophy), by accumulating lipids into their cytoplasm [26]. For that matter, fatty acid transporter proteins like FATP1 or the FAT translocase/CD36, together with lipoprotein lipase (LPL), are relevant actors that facilitate the fat uptake [27]. However, despite being the adipose tissue the largest body energy reserve, considered vital for the maintenance of energy homeostasis [28], its growth by hypertrophy has been associated with less responsive adipocytes to hormones and metabolites (i.e. insulin). This situation in humans derives in hypertrophic obesity and is closely linked to major health issues such as diabetes, hyperlipidemia or cardiovascular diseases [29], [30].

As indicated, the decision of MSCs fate can be affected by cell surrounding microenvironment and might be modulated by endocrine and dietary conditions. Moreover, the signals that induce adipogenesis, at the same time act as inhibitors of osteoblastogenesis, and *vice versa* [23]. Therefore, depending on the stimulus they receive, MSCs differentiate into one or another lineage. In mammals, low dietary n-3/n-6 ratios reduce bone formation and cause greater bone resorption [31], [32], [33], [34]. In the same way, changes in dietary fatty acids could modify the bone health and whole fat content due to this cellular interconversion, but although this is clear in mammals [33] it has not been proved in fish yet. Recently, culture models of MSCs have been established in fish from various adult tissues including fat and bone, and those MSCs have been demonstrated to hold the plasticity to differentiate into lineages different from the original tissue [18], [35], [36], [37] [38], [39].

In this context, the aim of the present work was to study the effects of the fatty acids EPA and DHA, present mainly in fish oil, and those of LA and ALA, common in vegetable oils (such as soybean, rapeseed and linseed oils), on fat deposition and the expression of both adipogenic- and osteogenic-related genes, in MSCs derived from gilthead sea bream vertebrae. The study hypothesis was that these fatty acids, supplemented in the media, could induce the differentiation of bone-derived MSCs toward the adipogenic *versus* the osteogenic lineage, potentially producing phenotypically different adipocytes depending on the fatty acid source.

## Materials and methods

### Animals and ethics statement

Gilthead sea bream (*Sparus aurata*) were obtained from the Tinamenor fish farm (Cantabria, Spain) and maintained in the animal facilities of the Faculty of Biology at the University of Barcelona. Sexually immature juveniles of an average weight of 30g were kept in 200 L fiberglass tanks under a 12 h light/12 h dark photoperiod and fed *ad libitum* twice daily with a commercial diet (Optibream, Skretting, Burgos, Spain). All animal handling procedures complied with the Guidelines of the European Union Council (86/609/EU) and were approved by the Ethics and Animal Care Committee of the University of Barcelona, (permit numbers CEEA 210/14 and DAAM 6759).

### Primary cultures of bone-derived MSCs and experimental design

Primary cultures of gilthead sea bream bone-derived MSCs were performed as previously described [35]. Briefly, three juvenile gilthead sea bream were used for each culture. The fish were sacrificed by a blow to the head, and bone-derived MSCs were isolated from a piece of vertebra by mechanical disruption and enzymatic (i.e. collagenase) digestion. After several washes, cells and small vertebra fragments were plated with growth medium (GM) consisting on Dulbecco’s Modified Eagle Medium (DMEM) containing 10% fetal bovine serum (FBS) and 1% antibiotic/antimycotic solution and supplemented with 19mM NaCl and 1% fungizone (Invitrogen Life Technologies, Alcobendas, Spain), in 10cm culture dishes. After 1week, the fragments were removed, and the attached cells collected with 0.25% trypsin–EDTA (Invitrogen Life Technologies) and plated into new 10cm plates with fresh GM. From here, the cells were routinely subcultured every time they reached about 70–80% confluence and used for a maximum of 10 passages.

To perform the experiments the cells were seeded at a density of 1×10^4^ cells/cm^2^ in 24 well plates for the viability test and the lipid quantification assay and in 6 well plates for the gene expression analyses. The fatty acids were applied 3–4 days after plating and the duration of the treatments were of 6 h for gene expression analyses, 24 h to determine viability and 6, 24, 48 and 72 h to quantify lipid accumulation. In all cases, two wells were used for each experimental condition. The fatty acids selected were the following: EPA and DHA since are the most abundant n-3 fatty acids in fish oil sources, and LA and ALA because are essential fatty acids that are present at a high percentage in vegetable oils commonly used in fish feeds (i.e. soya and rapeseed oils). The fatty acids obtained from Cayman Chemical Company (Michigan, USA), were first dissolved in ethanol and used at a final concentration of 200 μM both, individually and in all tested combinations unless stated otherwise. Final concentration of ethanol was very low (below 1%) and did not cause any negative effects in cell viability as confirmed in preliminary assays (ethanol concentrations up to 10% were tested for 24 h, S1 Fig). For the PPARγ antagonists experiment, two commonly used covalent PPARγ ligands T0070907 (2-Chloro-5-nitro-N-4-pyridinyl-benzamide) and GW9662 (2-Chloro-5-nitro-N-phenylbenzamide) were used. These two compounds are referred to as antagonists because they physically block ligand binding by covalently modifying the Cys285 located in an orthosteric pocket embedded in the ligand-binding domain, although they do not have comparable effects with regards to transcription [40], [41]. Both were obtained from Sigma-Aldrich (Tres Cantos, Spain), diluted with dimethyl sulfoxide (DMSO) and applied together with the fatty acids at a final concentration of 10 μM according to previous literature [42].

### MTT cell assay

The methylthiazolyldiphenyl-tetrazolium bromide (MTT) assay was used to evaluate cell viability as previously described elsewhere [35]. Briefly, cells from 3–4 independent cultures were incubated the last 3 h of the total 24 h treatment with a final concentration of 0.5 mg/mL of MTT. Then, cells were washed with PBS, resuspended in 250 μL of DMSO per well and absorbance was read immediately using a microplate reader (Infinite 200, Tecan). Cell viability values were obtained from the absorbance measured at 570 nm, with 680 nm as the reference wavelength.

### Oil Red O staining

Intracellular neutral lipid accumulation was analyzed by Oil red O (ORO) staining as explained in [35]. Briefly, cells were fixed with 10% formalin for 1 h, subsequently rinsed with PBS, stained with 0.3% ORO prepared in 36% tri-ethyl phosphate for 2 h, and then rinsed with distilled water. Quantification of cell lipid content was calculated as the absorbance measured at 490 nm divided by the read at 630 nm (Infinite 200, Tecan) corresponding to the protein content. The latter was obtained after Comassie blue staining for 1 h and dye extraction by incubation of the cells with 85% propylene glycol during 3 h at 60°C [35]. Data are presented as fold change relative to the control (n = 3). The staining effectiveness was evaluated with a Zeiss Axiovert 40C (Carl Zeiss Inc., Germany) inverted research grade microscope equipped with a Canon EOS 1000D digital camera (magnification 20x).

### RNA extraction and cDNA synthesis

The cells were lysed with a cell scraper and TRI Reagent (Applied Biosystems, Alcobendas, Spain) in a total volume of 1 mL per each two wells. Total RNA was extracted according to the manufacturer’s recommendations, dissolved in DEPC-treated water (RNase-free), quantified using a NanoDrop 2000 spectrophotometer (Thermo Scientific, Alcobendas, Spain) and stored at −80°C. To eliminate any residual genomic DNA, total RNA (1 μg) was treated with DNase I (Invitrogen, Alcobendas, Spain) and converted into cDNA using the Transcriptor First Strand cDNA Synthesis Kit (Roche, Sant Cugat del Valles, Spain), following the manufacturer’s instructions.

### Quantitative PCR analyses

To characterize the transcriptional profile occurring during the differentiation of bone-derived MSCs into adipocyte-like cells, key genes implicated in osteogenesis, adipogenesis and energy metabolism regulation were analyzed by real-time quantitative PCR (qPCR). The genes evaluated comprise the following: the transcription factor *runx2*, and ECM components: fibronectin 1a (*fib1a*), matrix Gla protein (*mgp*), *op* and *on* for the osteogenic genes. The transcription factors or nuclear receptors: *pparg*, retinoid X receptor (*rxr*) and *cebpb*; the enzymes: fatty acid synthase (*fas*), lipoprotein lipase (*lpl*) and hormone-sensitive lipase (*hsl*); fatty acid transporters: *cd36*, fatty acid transport protein 1 (*fatp1*) and fatty acid binding protein 11 (*fabp11*) for the adipogenic genes. In addition, elongation factor 1 alfa (*ef1a*), ribosomal protein S18 (*rps18*), and beta-actin (*b-actin*) were tested as reference genes.

qPCR was performed using a CFX384 thermocycler (Bio-Rad, El Prat de Llobregat, Spain) as previously described [38]. Each qPCR reaction was performed in triplicate in a total volume of 5 μL, containing 2.5 μL of the iTaq Universal SYBR Green supermix (Bio-Rad, El Prat de Llobregat, Spain), 2 μL of diluted cDNA, 0.125 μL of each primer (250 nM) (Table 1), and milliQ water. Samples were amplified as follows: 95°C for 3 min, and then 40 cycles of 95°C for 10 s, followed by annealing 60–68°C for 30 s (primer-dependent, Table 1), followed by a dissociation step from 55 to 95°C with a 0.5°C increase every 5 s. A standard curve with a dilution series of a cDNA sample pool was constructed to determine the qPCR efficiency of each primer pair (Table 1), which was calculated using the CFX Manager Software (Bio-Rad). To determine the overall performance of each qPCR assay three control samples were used: no template control (NTC), no reverse transcriptase control (RTC), and PCR control (PCR). Relative expression levels of the target genes were determined by the Pfaffl method [43], using correction for primer efficiencies and normalizing the quantification cycle (Cq) value of each gene, registered during the annealing step to that of *b-actin* and *rps18*, the most stable reference genes among the different conditions (P > 0.05) determined using the CFX Manager Software (Bio-Rad). Data were obtained from 4–6 independent cultures.

**Table 1:** Primers sequences.

Gene	Primer sequence (5'→3')	Tm (°C)	Efficiency (%)	Acc. Num.
*runx2*	**F:** ACCCGTCCTACCTGAGTCC	60	104.1	JX232063
	**R:** AGAAGAACCTGGCAATCGTC			
*pparg*	**F:** CGCCGTGGACCTGTCAGAGC	66	94.1	AY590304
	**R:** GGAATGGATGGAGGAGGAGGAGATGG			
*rxr*	**F:** CCCGGATGCAAAAGGTCTCT	60	99.7	-
	**R:** ATGCTCCAGACACTTGAGGC			
*cebpb*	**F:** ATGCGCAACTTGGAGACTCA	60	95.5	-
	**R:** GATTAGACAAGCGGCCCAGT			
*fib1a*	**F:** CGGTAATAACTACAGAATCGGTGAG	60	96.7	FG262933
	**R:** CGCATTTGAACTCGCCCTTG			
*mgp*	**F:** TGTGTAATTTATGTAGTTGTTCTGTGGCATCTCC	68	101.1	AY065652
	**R:** CGGGCGGATAGTGTGAAAAATGGTTAGTG			
*on*	**F:** GTGGTGGTTCAGGCAGGGATTCTCA	68	94.3	AY239014
	**R:** AGGAGGAGGTCATCGTGGAAGAGCC			
*op*	**F:** AAAACCCAGGAGATAAACTCAAGACAACCCA	68	91.9	AY651247
	**R:** AGAACCGTGGCAAAGAGCAGAACGAA			
*fas*	**F:** TGGCAGCATACACACAGACC	60	95.7	AM952430
	**R:** CACACAGGGCTTCAGTTTCA			
*lpl*	**F:** GAGCACGCAGACAACCAGAA	60	108.3	AY495672
	**R:** GGGGTAGATGTCGATGTCGC			
*hsl*	**F:** GCTTTGCTTCAGTTTACCACCATTTC	60	92.0	EU254478
	**R:** GATGTAGCGACCCTTCTGGATGATGTG			
*cd36*	**F:** GTCGTGGCTCAAGTCTTCCA	60	96.8	-
	**R:** TTTCCCGTGGCCTGTATTCC			
*fatp1*	**F:** CAACAGAGGTGGAGGGCATT	60	102.7	-
	**R:** GGGGAGATACGCAGGAACAC			
*fabp11*	**F:** CATTTGAGGAGACCACCGCT	60	107.5	-
	**R**: ACTTGAGTTTGGTGGTACGCT			
*b-actin*	**F:** TCCTGCGGAATCCATGAGA	60	106.9	X89920
	**R:** GACGTCGCACTTCATGATGCT			
*ef1a*	**F:** CTTCAACGCTCAGGTCATCAT	60	97.4	AF184170
	**R:** GCACAGCGAAACGACCAAGGGGA			
*rps18*	**F:** AGGGTGTTGGCAGACGTTAC	60	107.3	AM490061
	**R:** CTTCTGCCTGTTGAGGAACC			

Primers used for real-time quantitative PCR. F, forward primer; R, reverse primer; Tm, annealing temperature; Acc. Num., GenBank accession number.

### Statistical analyses

Data normality and homoscedasticity were assessed using Shapiro–Wilk and Levene’s test, respectively. Independent samples’ Student’s t-test was used for comparison between two groups (each experimental treatment *versus* the control). For multiple mean comparisons (among fatty acid treatments) of normal distributed data, one-way ANOVA was used followed by Tukey’s or Dunnett’s T3 *post hoc* tests in case of homogeneous or heterogeneous variance data, respectively. When data did not fit normal distribution, the non-parametric Kruskal–Wallis test, followed by Mann–Whitney test, were used. Statistical analyses were performed using SPSS Statistics version 20 (IBM, Armonk, NY, USA). Results are presented as mean ± SEM. P < 0.05 was considered to indicate a statistically significant difference. Graphs were generated using GraphPad Prism version 6.00 for Windows (GraphPad Software, La Jolla, CA, USA, www.graphpad.com).

## Results

### Fatty acids effects in cell viability and differentiation

**Fig 1:**
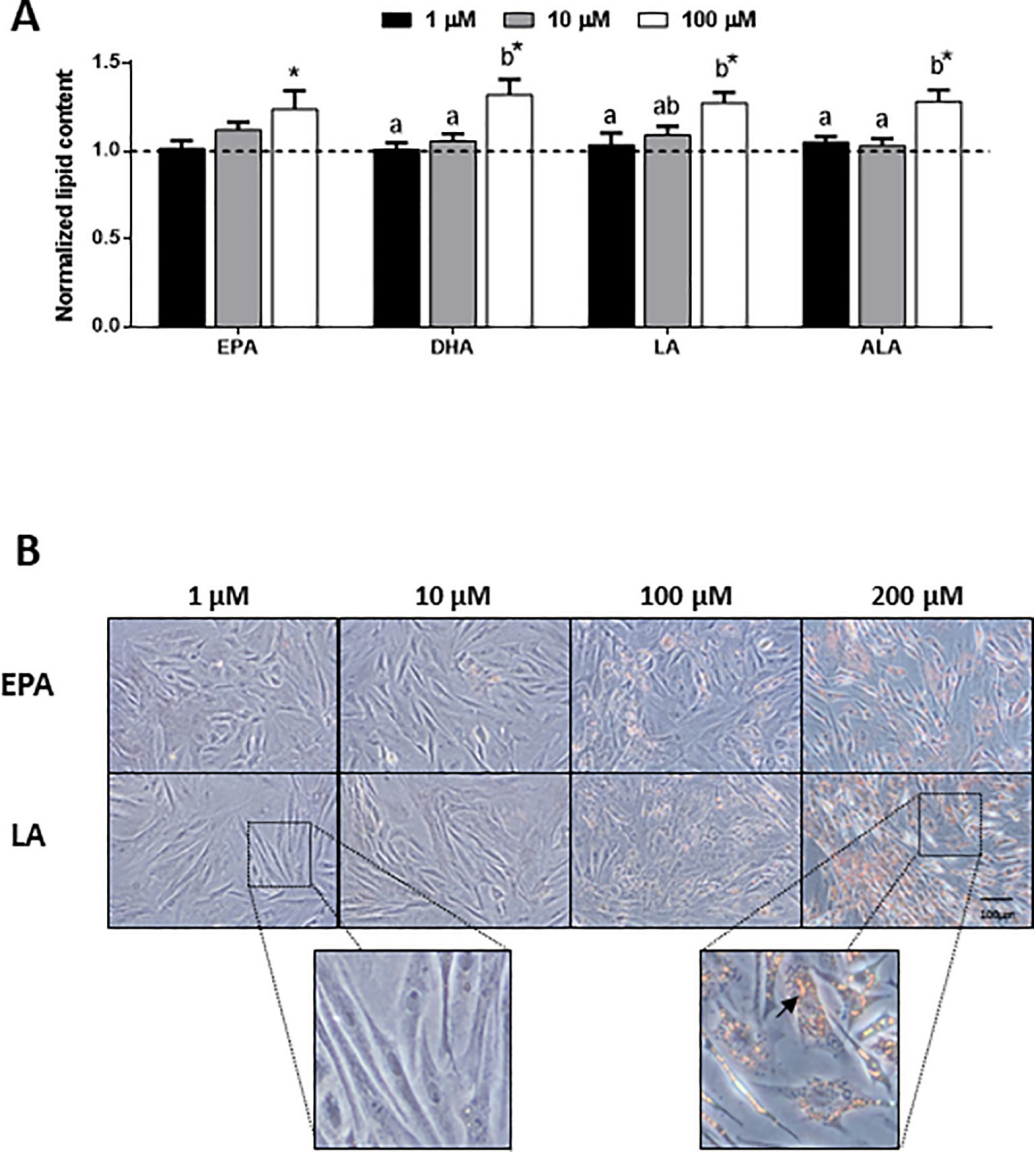
Dose response of fatty acids on lipid accumulation. (A) Quantification of lipid content normalized by protein and (B) representative phase-contrast images of gilthead sea bream bone-derived cells after staining with Oil red O. Cells were treated at day 4 with different concentrations of individual fatty acids, or were left untreated as control (dashed line in A) for 48 h. In (A) data are shown as mean + SEM (n = 3–4). Significant differences (p<0.05) among concentrations are indicated by different letters. Asterisks indicate significant differences (p<0.05) with the control. In (B) magnification 20x and enlarged views, arrow indicates lipid droplets. EPA: eicosapentaenoic acid; DHA: docosahexaenoic acid; LA: linoleic acid; ALA: α-linolenic acid.

Preliminary analyses demonstrated that cell viability was unaffected by the addition of the different fatty acids up to the 200 μM concentration tested (S1 Fig). On the other hand, a dose response was observed with regards to lipid accumulation upon a 48 h treatment with each one of the four fatty acids (EPA, DHA, LA and ALA), showing at the 100 μM concentration significantly higher intracellular lipid content compared to lower doses and the control condition without fatty acids (Fig 1A). Moreover, the images obtained after ORO staining of the cells upon all treatments (EPA and LA shown in Fig 1B as a representation) confirmed this observation, being the 200 μM concentration the one causing higher lipid accumulation and therefore, the one selected for the following experiments. In addition, we could observe in these images the change of cell morphology in response to the treatments, becoming the cells more rounded with an enlarged cytoplasm while losing the fibroblastic shape of MSCs.

**Fig 2:**
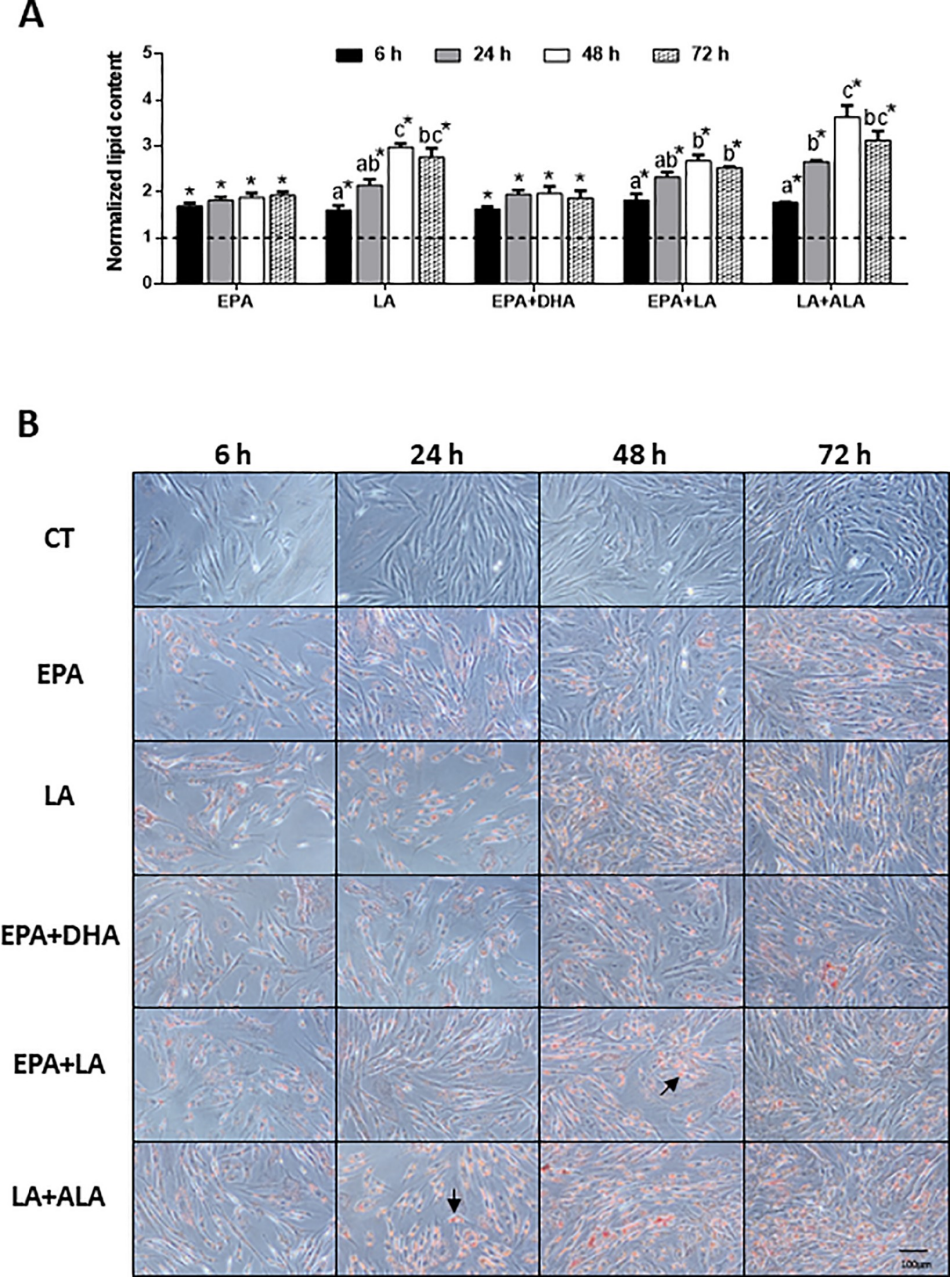
Time course of lipid accumulation by fatty acids. (A) Quantification of lipid content normalized by protein and (B) representative phase-contrast images of gilthead sea bream bone-derived cells stained with Oil red O. Cells were treated at day 4 with selected individual or combined fatty acids (200 μM), or were left untreated as control (CT, dashed line in A) for 6, 24, 48 and 72 h. In (A) data are shown as mean + SEM (n = 3–4). Significant differences (p<0.05) among time-points are indicated by different letters. Asterisks indicate significant differences (p<0.05) with the control. In (B) magnification 20x, arrows indicate lipid droplets. EPA: eicosapentaenoic acid; DHA: docosahexaenoic acid; LA: linoleic acid; ALA: α-linolenic acid.

To further determine the effects through time of selected fatty acids on lipid accumulation, individual treatments with EPA and LA as representatives from the highly present fatty acids in fish and vegetable oils respectively, plus the mixtures EPA+DHA as the fish oil combination, LA+ALA as the vegetable oils one and EPA+LA as the combination containing one fatty acid of each source, were tested at 6, 24, 48 and 72 h. All treatments caused a significant increase in cell lipid content compared to the control condition. Moreover, a time-dependent response up to 48 h (remaining high at 72 h) was also observed by treatments including LA alone or in combination (Fig 2A). The effects of these fatty acids inducing cell lipid accumulation and differentiation (i.e. rounding up) compared to the control condition were confirmed by the microscopic visual evaluation of the culture (Fig 2B). According to these results, the 6 h treatment was selected to evaluate gene expression in subsequent experiments, in order to observe the immediate effects of these fatty acids at a transcriptional level inducing changes on cell metabolism.

### Fatty acids effects in gene expression

**Fig 3:**
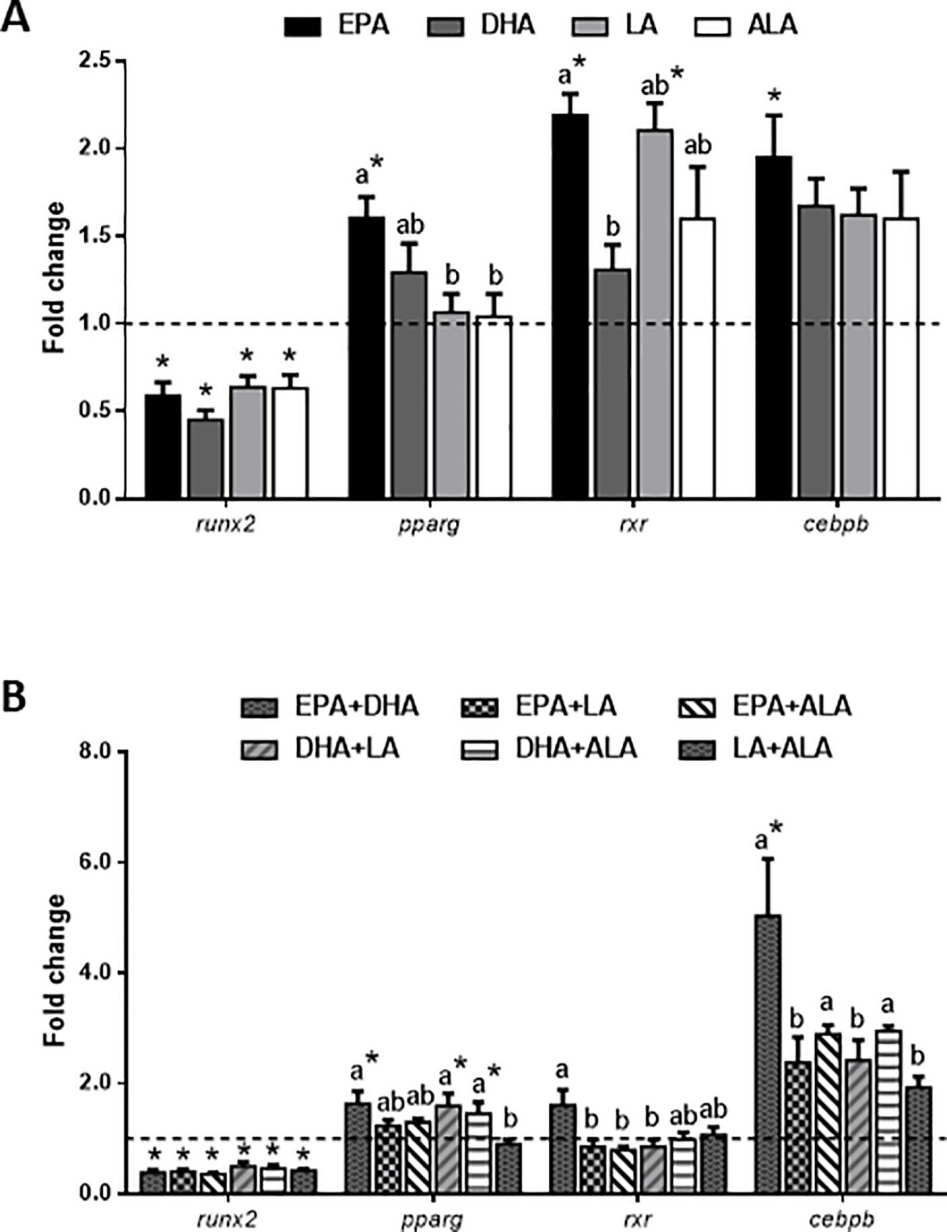
Fatty acids effects on transcription factors gene expression. Relative expression of genes related to the processes of osteogenesis (*runx2*) and adipogenesis (*pparg*, *rxr* and *cebpb*) normalized to *b-actin* and *rps18* in gilthead sea bream bone-derived cells. Cells at day 4 were treated with different (A) individual or (B) combined fatty acids or were left untreated as control (dashed lines) for 6 h. Data are shown as mean + SEM (n = 3–4). Significant differences (p<0.05) among treatments are indicated by different letters. Asterisks indicate significant differences (p<0.05) with the control. EPA: eicosapentaenoic acid; DHA: docosahexaenoic acid; LA: linoleic acid; ALA: α-linolenic acid.

The gene expression of *runx2*, the key transcription factor of the osteogenic process, was significantly down-regulated by all fatty acids, either applied individually or combined, compared to the control condition, although differences were not observed among treatments (Fig 3A and 3B). Contrarily, the principal genes involved in the first steps of adipogenesis, were up-regulated after EPA treatment, significantly for *pparg*, *cebpb* and *rxr*, and the latter also by LA treatment, when compared to the control. In addition, EPA-treated cells showed significant differences with respect to those treated with LA or ALA for *pparg* gene expression and with DHA as well in the case of *rxr* (Fig 3A). Furthermore, the different combinations caused patterns of expression for these genes according to the fatty acids included in the mixture (Fig 3B). Namely, the combinations containing one (i.e. DHA) or specially the two fatty acids present in fish oils, significantly up-regulated the transcript levels of the adipogenic genes *pparg* and *cebpb*, compared to the control condition and the combination of LA+ALA. In addition, the mRNA levels of *rxr* and *cebpb* were significantly lower in response to the combinations containing LA (especially in the one of LA+ALA), respect to the EPA+DHA mixture (Fig 3B).

**Fig 4:**
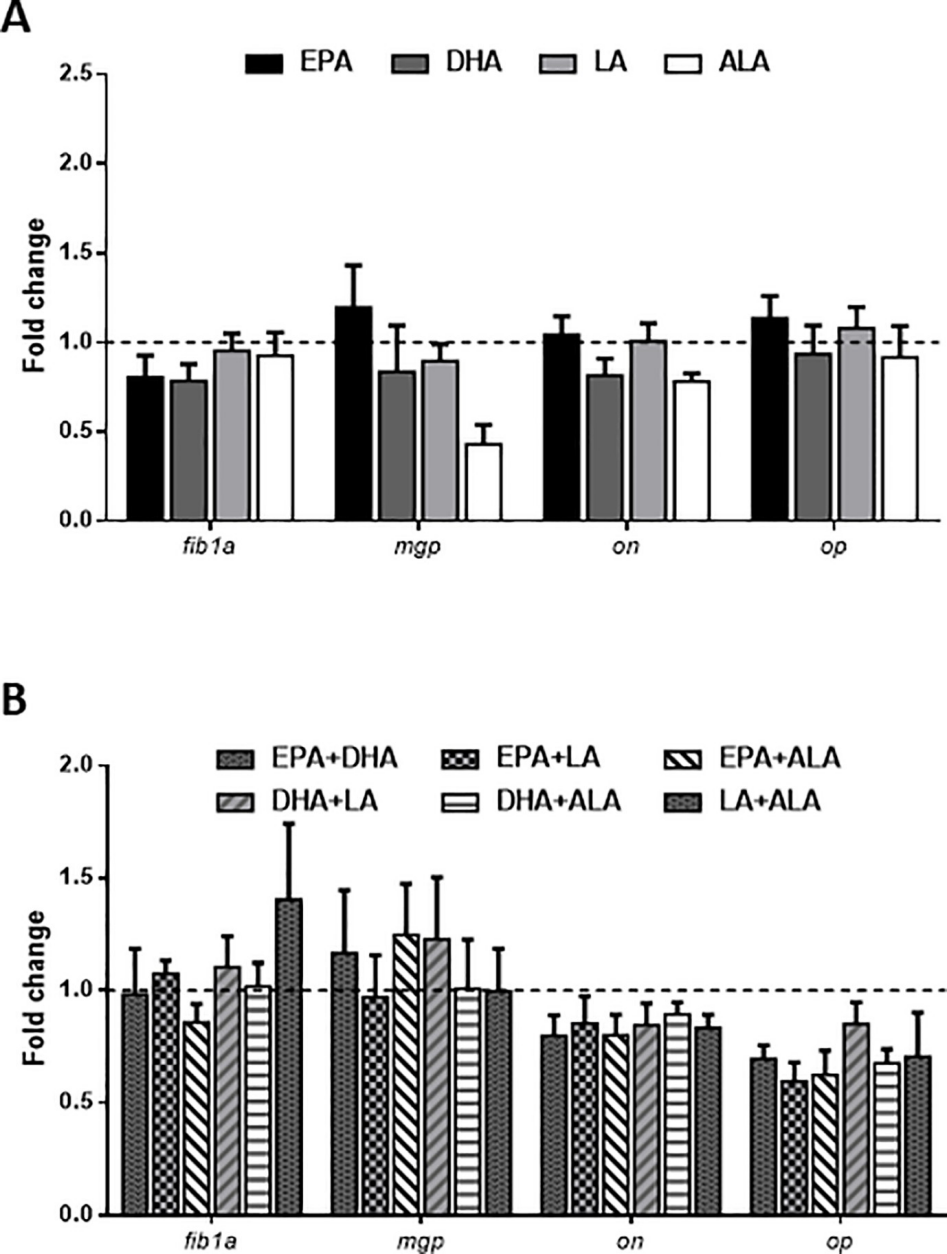
Fatty acids effects on osteogenic genes expression. Relative expression of genes related to the process of osteogenesis (*fib1a*, *mgp*, *on* and *op*) normalized to *b-actin* and *rps18* in gilthead sea bream bone-derived cells. Cells at day 4 were treated with different (A) individual or (B) combined fatty acids or were left untreated as control (dashed lines) for 6 h. Data are shown as mean + SEM (n = 3–4). Significant differences (p<0.05) among treatments are indicated by different letters. Asterisks indicate significant differences (p<0.05) with the control. EPA: eicosapentaenoic acid; DHA: docosahexaenoic acid; LA: linoleic acid; ALA: α-linolenic acid.

**Fig 5:**
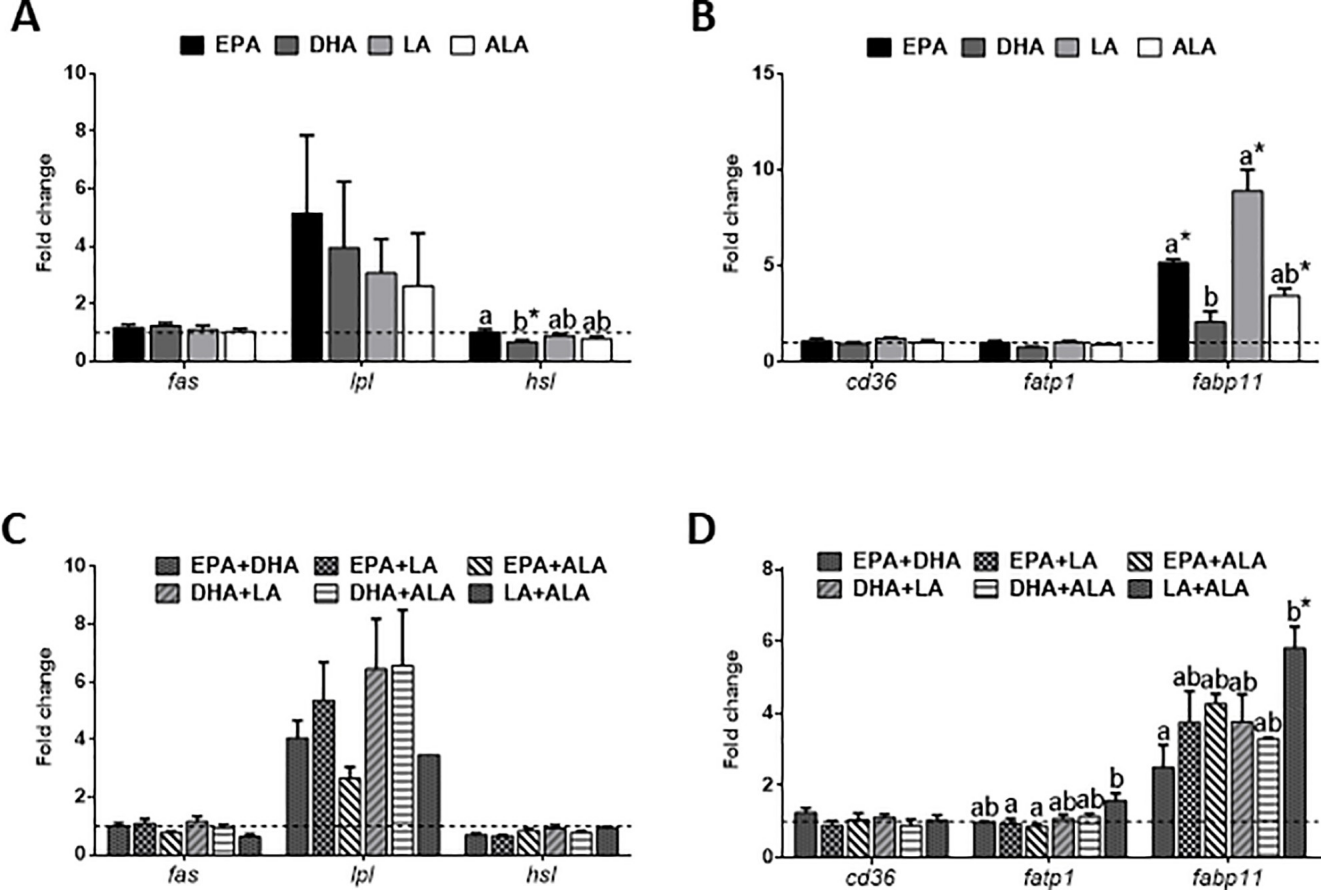
Fatty acids effects on adipogenic genes expression. Relative expression of genes related to lipid metabolism, including (A, C) enzymes (*fas*, *lpl* and *hsl*) and (B, D) fatty acid transporters (*cd36*, *fatp1* and *fabp11*) normalized to *b-actin* and *rps18* in gilthead sea bream bone-derived cells. Cells at day 4 were incubated with different (A, B) individual or (C, D) combined fatty acids, or were left untreated as control (dashed lines) for 6 h. Data are shown as mean + SEM (n = 3–4). Significant differences (p<0.05) among treatments are indicated by different letters. Asterisks indicate significant differences (p<0.05) with the control. EPA: eicosapentaenoic acid; DHA: docosahexaenoic acid; LA: linoleic acid; ALA: α-linolenic acid.

The expression analysis of osteogenic genes involved in ECM formation and/or mineralization showed how these remained unaltered in cells in the presence of fatty acids either applied alone or in combination. Nevertheless, the treatments with two fatty acids combined caused *on* and *op* to have a lower, but not significant expression, compared to the control (Fig 4A and 4B). Moreover, genes encoding lipid metabolism-related enzymes and fatty acid transporters were studied to unravel whether a possible regulation of pro-adipogenic genes was related to the process of differentiation of MSCs into adipocyte-like cells. EPA treatment caused an increase in *hsl* mRNA levels, although only significant when compared to DHA-treated cells, while *fas* and *lpl* remained stable (Fig 5A). Concerning the fatty acid transporters, EPA, LA and ALA significantly up-regulated the mRNA levels of *fabp11* compared to the control (Fig 5B). Even applying combinations of the different fatty acids to the cells, the gene expression of *fas*, *lpl*, *hsl* and *cd36* remained unaffected (Fig 5C and 5D). Nevertheless, the combination of the two fatty acids more common in vegetable oils (LA+ALA), significantly up-regulated the transcript expression of *fabp11* in comparison to the combination with the fatty acids EPA+DHA and the control condition. On the other hand, *fatp1* levels were significantly higher in response to LA+ALA when compared to the combinations containing EPA and either one of these two fatty acids from vegetable oils, but not with the control (Fig 5D).

### Effects of PPARγ antagonists in gene expression

**Fig 6:**
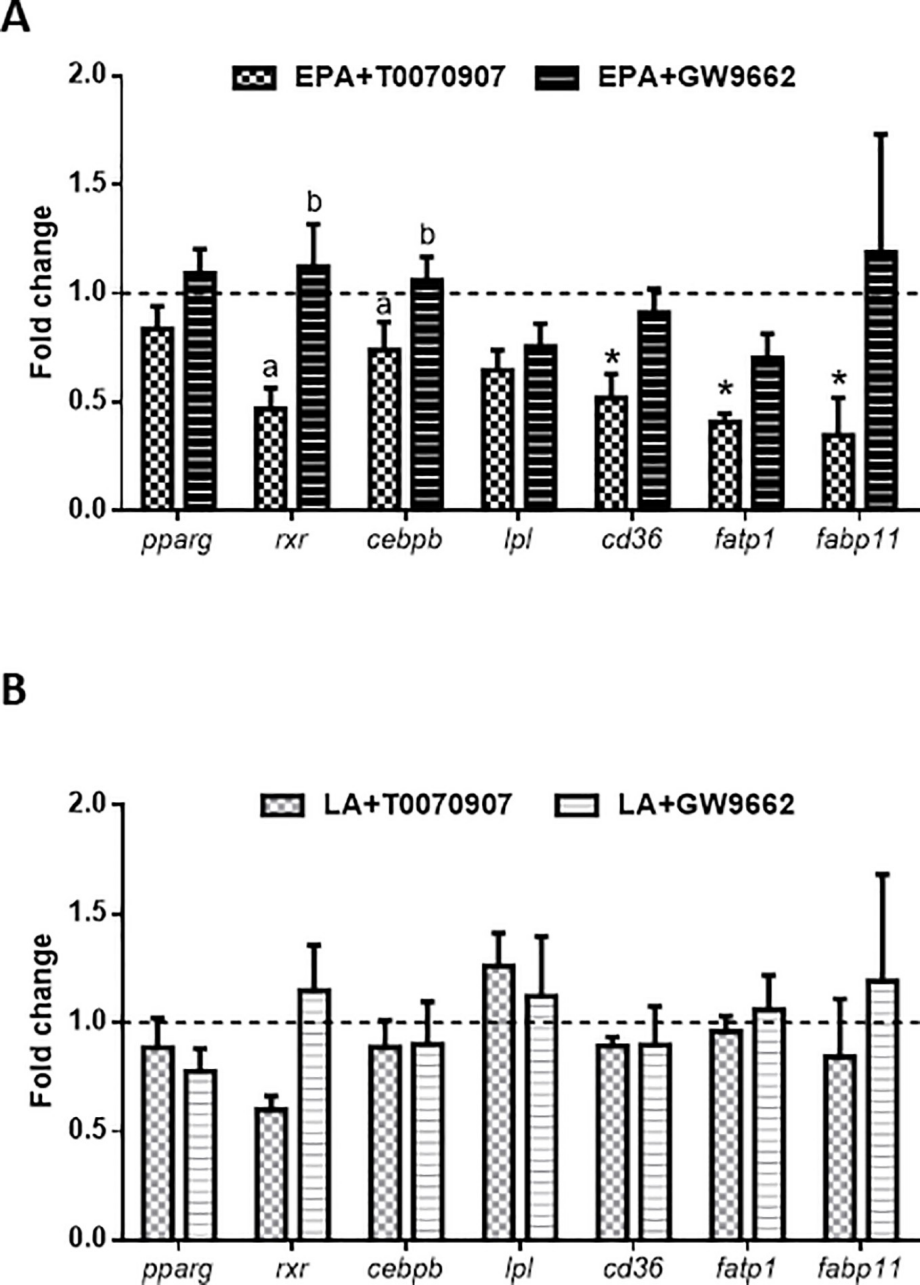
PPARγ antagonists effects in adipogenic genes expression. Relative expression of genes related to adipogenesis and lipid metabolism (*pparg*, *rxr*, *cebpb*, *lpl*, *cd36*, *fatp1* and *fabp11*) normalized to *b-actin* and *rps18* in gilthead sea bream bone-derived cells. Cells at day 4 were incubated with the fatty acids (A) EPA or (B) LA in the absence (dashed line, used as control) or presence of a PPARγ antagonist (T0070907 or GW9662) for 6 h. Data are shown as mean + SEM (n = 3–4). Significant differences (p<0.05) among treatments are indicated by different letters. Asterisks show significant differences (p<0.05) with the control. EPA: eicosapentaenoic acid; LA: linoleic acid.

Two different antagonists of PPARγ were applied to cells treated with either EPA or LA, to elucidate the potential different mechanism of action of these fatty acids inducing the differentiation of the bone-derived MSCs into adipocyte-like cells. First, viability assays were performed to assure non-toxicity of the products (S1 Fig). Next, taking into account that the transcriptional effects of each fatty acid alone in comparison to the control were already reported, the condition of each fatty acid in the absence of antagonists was used as the corresponding control in this set of experiments. Cells treated with EPA and T0070907 showed an overall decrease in expression for the genes studied, which was significant in comparison to the treatment with the other antagonist, GW9662 for *rxr* and *cebpb* and, to the control condition for the fatty acids transporters *cd36*, *fatp1* and *fabp11* (Fig 6A). Contrarily, the cells treated with LA and either one of the two antagonists, did not show any significant changes in gene expression (Fig 6B).

## Discussion

This study has focused on the characterization of the likely differential effects of fatty acids typical from fish oil (EPA and DHA) and those most commonly found in vegetable oils (LA and ALA) on cellular plasticity and metabolism. To this end, we used as a model an *in vitro* culture of MSCs derived from vertebra bone of gilthead sea bream (*S*. *aurata*), one of the most cultivated species in Mediterranean aquaculture. The main objective was to evaluate the lineage-induction potential over the MSCs of these fatty acids, not only for its possible relevance in fish nutrition and welfare, but also to validate the cell system to further study the multipotentiality of piscine MSCs and their regulation.

The first step was to check that the incubation with the fatty acids was not causing any deleterious effect on the cells. Thus, an MTT assay was run showing that none of the fatty acids significantly affected bone-derived MSCs viability at concentrations up to 200 μM, although a slight rise in viability could be seen with the 100 μM concentrations, maybe related to the increased metabolic activity of the cells along the induced process of differentiation. These results coincide with those in another study, where fatty acid treatments were performed on human MSCs grown in osteogenic media, to see their possible effect on osteoblastogenesis [44]. Also, cell viability was verified after applying different fatty acids (e.g. EPA, DHA and arachidonic) at a 100 μM concentration on the skeletal VSa16 cell line of gilthead sea bream, demonstrating that such treatments can stimulate proliferation without signs of toxic effects [45].

Bone marrow MSCs in mammals retain a high degree of plasticity and their fate is affected by cell culture medium composition [23]. In the present study, accumulation of lipid content induced by fatty acids added to the media was dose-dependent as confirmed through quantification and microscopy observation of the morphological changes, and the intensification of the red aura occupied in the cells by ORO staining. Previously, the ability of MSCs derived from bone to differentiate, in addition to osteoblasts, into adipocyte-like cells was demonstrated, by applying adipogenic media containing two different concentrations of lipid mixture; thus, confirming their multipotentiality [35]. The presence of fatty acids in such specific media seems to be critical to shoot the adipogenic process in undetermined fish cells [37], [46], [47], [48], and this has also been observed in avian adipocyte precursor cells [49]. Accordingly, in the present study, the cells began to accumulate lipids potentially inducing adipocyte-like differentiation in response to the fatty acid treatments. However, LA and the combinations containing this fatty acid were those that produced a greater effect, whereas the combination of the two fatty acids mainly present in fish oil (EPA+DHA) caused lower lipid deposition. Similarly, in mature salmon adipocytes it was observed that fatty acids from vegetable oils (i.e. oleic acid) were able to induce more triacylglycerol accumulation than fatty acids characteristic of fish oils [50]. Other studies have also shown this lower capacity of fatty acids from fish oils to be stored in adipose cells, such as those performed on 3T3-L1 pre-adipocytes, where DHA reduced dose-dependently fat deposition likely by suppressing lipid filling [51]. Overall, the data suggest that the n-6 PUFA LA may stimulate the uptake and depot of extracellular fats in these adipocyte-like cells, more than the other treatments tested.

Next, to further evaluate the effects of fatty acids on MSCs lineage determination, expression of relevant driving genes was analyzed. *runx2* codifies for a key transcriptional activator that promotes osteoblastogenesis thus, inhibiting the determination and subsequent differentiation of MSCs into other cell lineages [24]. On the other hand, PPARγ, a member of the hormone nuclear receptors family, after interaction with specific ligands such as LC-PUFA, activates the transcription of genes involved in adipogenesis and lipid metabolism determining the adipocyte phenotype of MSCs [25]. Moreover, induction of *pparg* expression can result in the inhibition of differentiation toward osteoblasts, as it has been described in some mammalian studies by acting as a suppressor of *runx2* [23], [52], [53]. Besides, overexpression of *runx2* in rat MSCs derived from adipose tissue produces a decrease in the expression of *pparg* [54]; consequently, these two transcription factors seem to act by negatively regulating each other. In Atlantic salmon, *pparg* is also silenced when the culture medium used is osteogenic, while *runx2* has its expression inhibited in the presence of an adipogenic medium [39]. In agreement with these observations, in our study, all fatty acid treatments, either alone or in combination, down-regulated *runx2*, although only EPA was able to significantly increase *pparg* gene expression. In fact, when treating the gilthead sea bream osteoblast-like VSa16 cell line with EPA, a decrease in the mRNA levels of *runx2* was also reported [45]. Accordingly, an increase in the transcript levels of *pparg* was also caused in human MSCs due to EPA treatment [44]. Despite significant differences were not observed with DHA alone, up-regulation of this key adipogenic gene was found with all combinations containing this fatty acid. Interestingly, both EPA and DHA had been considered for years, natural ligands of *pparg*, and to have greater potency on activating this transcription factor, compared to the n-6 PUFA (i.e. LA) [55]; so, these findings propose a direct effect of these n-3 LC-PUFA stimulating adipogenesis, as previously described in mammalian models [25]. Furthermore, *cebpb* expression was also up-regulated in response to EPA and by the combination EPA+DHA, supporting that the initiation of the adipogenic process is taking place upon those treatments. In fact, at least in mammals, *cebpb* contributes to stimulate *pparg* expression during early adipogenesis [56], [57]. Moreover, similar results were found when the gene expression of *rxr* was analyzed, since EPA, but also LA, could significantly up-regulate it. The nuclear receptor RXR forms a heterodimer among others with PPARγ, to regulate the transcription of genes related to lipid metabolism, thus also driving adipocyte differentiation [58]. Nevertheless, knowledge on the PPARγ-RXR heterodimers, as well as their response to fatty acids in fish is very limited.

To further evaluate if the bone-derived MSCs are deviated from the osteogenic process when the fatty acid treatments are applied, the expression of various genes related to both early osteogenesis and late mineralization of bone ECM was determined [36]. The mRNA levels of *fib1a*, *mgp*, *on*, and *op* remained constant in response to all the treatments, similarly as in [38], in the same cell model after addition of a standard adipogenic medium. With these results, we could suggest that the osteogenic process, to which these cells were previously predestined in their tissue microenvironment, has stopped. This deregulation of MSCs determination and/or trans-differentiation has been related not only in mammals, but also in fish, with developmental disorders or disease states, such as distraction osteogenesis in rats [59], bone loss in osteoporotic human patients [60] and reduced or malformed vertebrae in Atlantic salmon [61], [62], [63], [64], in which, diet specifically, has been shown as a causative factor [65].

Regarding expression of adipocyte markers, specifically genes that codify for key enzymes such as *fas*, involved in the synthesis of fatty acids, remained stable, maybe due to a direct inhibition of *de novo* synthesis caused by the addition of fatty acids into the culture medium. Similarly, differences could not be found in *lpl* or *hsl* gene expression during differentiation into adipocytes of rainbow trout cultured pre-adipocytes when the whole transcriptional profile of this process was analyzed [66]. Nevertheless, increased gene expression in the late phases of adipocyte differentiation has been reported for *lpl* in red sea bream [47] and Atlantic salmon [50].

Concerning the genes involved in the uptake and transport of fatty acids, *cd36* is, among others, a target gene of PPARγ [25]. In this context, PPARγ activation was found to induce *cd36* expression and adipocyte differentiation of the arterial rat VSMCs line [67]. In our study, the increased *pparg* mRNA levels at 6 h incubation in response to EPA, but not in *cd36*, suggest a possible delayed up-regulation of the latter. Furthermore, in Atlantic salmon and rainbow trout, the mRNA levels of *fatp1* increased during the differentiation of pre-adipocytes, at earlier stages than *fabp11*, indicating that the former has an important role in the induction of adipogenesis and in the uptake of fatty acids from the environment [27], [68]. In our study, the combination of LA+ALA or LA alone caused an increase in *fatp1* and *fabp11* mRNA levels compared to other combinations. Altogether, these results indicated a more direct stimulation of fat transporters expression by fatty acids of vegetal origin, especially LA, suggesting a possible mechanism of action to induce adipogenesis through enhancing fatty acid uptake. These observations together with the elevated capacity of lipid accumulation when cells were treated with LA are in agreement with a previous study in gilthead sea bream, in which diets with high content in vegetable oils induced adipocyte hypertrophy [12]. On the other hand, DHA and more remarkably EPA, showed a higher potential to stimulate adipogenesis via up-regulation of *pparg* gene expression, thus inducing the adipocyte-like phenotype but with lower lipid content. Hence, since smaller adipocytes have the metabolic advantage of retaining insulin sensitivity and protect other tissues from lipotoxicity according to works in mammals [69], further studies would be of great interest to demonstrate if fish oil-derived fatty acids would lead to healthier cells as well in fish.

To corroborate the differential action of EPA and LA driving adipocyte-like development of bone-derived MSCs through PPARγ activation, their effects in combination with two PPARγ antagonists were evaluated. According to the results, LA action was unaffected, demonstrating the direct effect of this fatty acid on the transport of lipids due to *fabp11* and *fatp1* up-regulated gene expression. With regards to EPA treatment, the antagonist GW9662 did not cause any change, but the use of the specific antagonist T0070907, triggered a remarkable down-regulation on transcript levels of the three fatty acid transporters and the factors *rxr* and *cebpb*, although not of *pparg*. Accordingly, in mammals GW9662 shows negligible effects on transcription compared to T0070907, which displays properties of an inverse agonist, showing effects on transcription opposite to well-known PPARγ agonists such as [40], [41]. Similarly, the antiobesogenic effect of these antagonists has been shown in zebrafish larvae *in vivo*, although without performing transcriptomic analyses [42], [70]. Overall, the current data would suggest an inhibition of the adipogenic process, including lipid internalization, mediated at least in part by the incapability of EPA to activate PPARγ and the companion transcription factors. In agreement with this hypothesis, other authors using this same antagonist showed a decrease in *cd36* mRNA levels not depending on the increased gene expression of *pparg*, but elevated PPARγ activity [71]. Thus, blockage of EPA action by T00709007 indicates that via the action of this transcription factor, EPA may also up-regulate fat transporters to ultimately stimulate adipocyte differentiation of MSCs. These results agree with the capacity of n-3 LC-PUFA to promote the formation of healthy new adipocytes [72]. An example of a similar scenario may be the antidiabetic treatment with the full PPARγ agonists, the thiazolidinediones (i.e. troglitazone or pioglitazone), which have been shown, at least in rodent models, to favor remodeling of the adipose tissue by promoting pre-adipocyte recruitment for hyperplastic growth [73], [74].

## Conclusions

**Fig 7:**
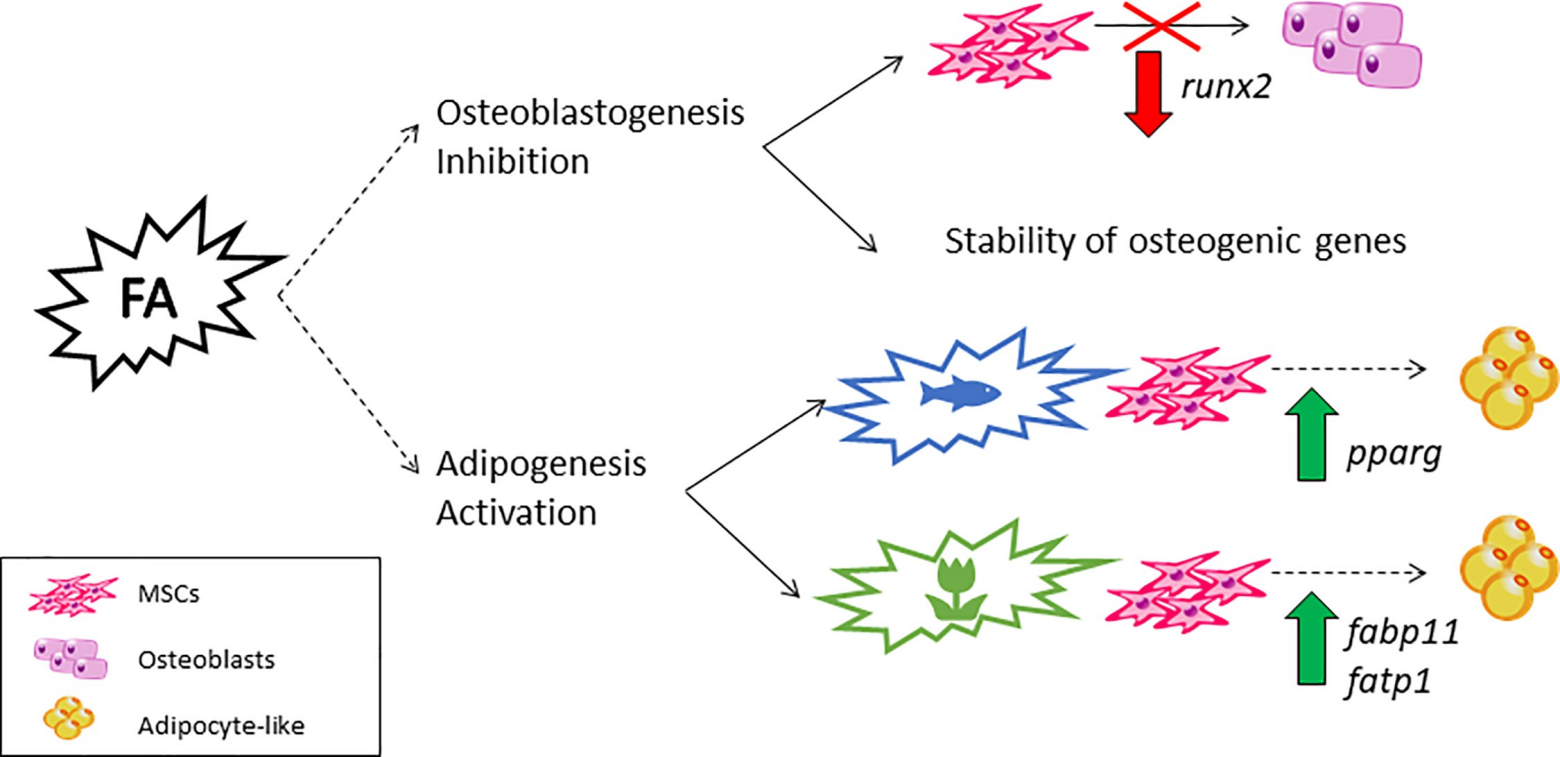
Summary of fatty acids effects on gilthead sea bream bone-derived cells. Schematic representation summarizing the effects of fatty acid (FA) treatments over bone-derived mesenchymal stem cells (MSCs) of gilthead sea bream. The fatty acids produce an inhibition of the osteogenic process through causing a down-regulation of *runx2* and a stabilization of the osteogenic genes relative expression. On the other hand, fatty acids induce adipogenesis, with those fatty acids characteristic from fish oils apparently via up-regulating *pparg* mRNA levels and in contrast, those typical from vegetable oils increasing the relative gene expression of fatty acid transporters (*fabp11* and *fatp1*), thus potentially enhancing cell lipid accumulation.

Gilthead sea bream bone-derived MSCs treated with one or two combined fatty acids undergo morphological and transcriptional changes, increasing lipid accumulation as well as the expression of adipogenic genes while decreasing or maintaining stable those related to the osteogenic process. This confirms the plasticity of these cells and supports their use as a model to study MSCs fate modulation. Besides, these findings should be also considered when studying fish bone structure and function, since at least in humans, there is a correlation between the appearance of bone marrow fat and the reduced bone forming capacity observed during diabetes and aging [75], [76]. Our data also suggest that fatty acids might be inducing adipogenesis potentially through different pathways, with fish oil-derived fatty acids such as EPA causing mainly formation of new adipocytes through activation of PPARγ, whereas vegetable fatty acids like LA appear to rather induce a process of fat accumulation in committed pre-adipocytes (Fig 7). These results advise that fatty acids from plant origin should be wisely used in aquafeeds, as they could induce the formation of less sensitive and functional hypertrophic adipocytes as previously suggested [12]. While we should be cautious because most of our data is based on a transcriptional level, and further studies are required to validate these observations; overall, this needs to be considered in feeds formulation to carefully find a balance according to the nature of the oil sources to ensure a healthy and high-quality fish.

## Supporting information

S1 Fig Effects of ethanol, fatty acids and antagonists treatments on cell viability. Viability of gilthead sea bream bone-derived cells at day 4 determined by means of the MTT assay. Cells were treated (**A**) for 24 h with different concentrations of ethanol; or for 6 h (**B**) with different concentrations of selected fatty acids (EPA and LA) or were left untreated as control (dashed line), and (**C**) with the fatty acids EPA or LA in the absence (dashed line) or the presence of a PPARγ antagonist (T0070907 or GW9662). Data are shown as mean + SEM (n = 3). Significant differences (p<0.05) among concentrations are indicated by different letters. Asterisks indicate significant differences (p<0.05) with the corresponding control. EPA: eicosapentaenoic acid; LA: linoleic acid.
(TIF)

S1 File Submit R1.zip contains the files with the complete raw data. (ZIP)

## References

[1] Pike IH, Andrew J. Fish oil: production and use now and in the future. Lipid Technology, 2010; 22, 59–61.

[2] Shahidi F, Ambigaipalan P. Omega-3 Polyunsaturated Fatty Acids and Their Health Benefits. Annual Review of Food Science and Technology, 2018; 9(1), 345–381. 10.1146/annurev-food-111317-095850 29350557

[3] Watanabe T. Strategies for further development of aquatic feeds. Fisheries Science, 2002; 68(2), 242–252. 10.1046/j.1444-2906.2002.00418.x

[4] Miller MR, Nichols PD, Carter CG. n-3 oil sources for use in aquaculture–alternatives to the unsustainable harvest of wild fish. Nutrition Research Reviews, 2008; 21(02), 85 10.1017/S0954422408102414 19087364

[5] Tocher DR. Metabolism and functions of lipids and fatty acids in teleost fish. Reviews in Fisheries Science, 2003; 11(2), 107–184. 10.1080/713610925

[6] Bell JG, McEvoy J, Tocher DR, McGhee F, Campbell PJ, Sargent JR. Replacement of fish oil with rapeseed oil in diets of Atlantic salmon (Salmo salar) affects tissue lipid compositions and hepatocyte fatty acid metabolism. The Journal of Nutrition, 2001; 131(5), 1535–1543. 10.1093/jn/131.5.1535 11340112

[7] Bell JG, Henderson RJ, Tocher DR, McGhee F, Dick JR, Porter A, et al Substituting fish oil with crude palm oil in the diet of Atlantic salmon (Salmo salar) affects muscle fatty acid composition and hepatic fatty acid metabolism. The Journal of Nutrition, 2002; 132(2), 222–230. 10.1093/jn/132.2.222 11823582

[8] Benedito-Palos L, Navarro JC, Sitjà-Bobadilla A, Gordon Bell J, Kaushik S, Pérez-Sánchez J. High levels of vegetable oils in plant protein-rich diets fed to gilthead sea bream (Sparus aurata L.): growth performance, muscle fatty acid profiles and histological alterations of target tissues. British Journal of Nutrition, 2008; 100(05), 992 10.1017/S0007114508966071 18377678

[9] Benedito-Palos L, Navarro JC, Bermejo-Nogales A, Saera-Vila A, Kaushik S, Pérez-Sánchez J. The time course of fish oil wash-out follows a simple dilution model in gilthead sea bream (Sparus aurata L.) fed graded levels of vegetable oils. Aquaculture, 2009; 288(1–2), 98–105. 10.1016/J.AQUACULTURE.2008.11.010

[10] Benedito-Palos L, Navarro JC, Kaushik S, Pérez-Sánchez J. Tissue-specific robustness of fatty acid signatures in cultured gilthead sea bream (Sparus aurata L.) fed practical diets with a combined high replacement of fish meal and fish oil1. Journal of Animal Science, 2010; 88(5), 1759–1770. 10.2527/jas.2009-2564 20081079

[11] Bouraoui L, Sánchez-Gurmaches J, Cruz-Garcia L, Gutiérrez J, Benedito-Palos L, Pérez-Sánchez J, Navarro I. Effect of dietary fish meal and fish oil replacement on lipogenic and lipoprotein lipase activities and plasma insulin in gilthead sea bream (Sparus aurata). Aquaculture Nutrition, 2011; 17(1), 54–63. 10.1111/j.1365-2095.2009.00706.x

[12] Cruz-Garcia L, Sánchez-Gurmaches J, Bouraoui L, Saera-Vila A, Pérez-Sánchez J, Gutiérrez J, Navarro I. Changes in adipocyte cell size, gene expression of lipid metabolism markers, and lipolytic responses induced by dietary fish oil replacement in gilthead sea bream (Sparus aurata L.). Comparative Biochemistry and Physiology Part A: Molecular and Integrative Physiology, 2011; 158(4), 391–399. 10.1016/j.cbpa.2010.11.024 21130894

[13] Montero D, Kalinowski T, Obach A, Robaina L, Tort L, Caballero M, et al Vegetable lipid sources for gilthead seabream (Sparus aurata): effects on fish health. Aquaculture, 2003; 225(1–4), 353–370. 10.1016/S0044-8486(03)00301-6

[14] Montero D, Mathlouthi F, Tort L, Afonso JM, Torrecillas S, Fernández-Vaquero A, et al Replacement of dietary fish oil by vegetable oils affects humoral immunity and expression of pro-inflammatory cytokines genes in gilthead sea bream Sparus aurata. Fish and Shellfish Immunology, 2010; 29(6), 1073–1081. 10.1016/j.fsi.2010.08.024 20817101

[15] Villeneuve L, Gisbert E, Le Delliou H, Cahu CL, Zambonino-Infante JL. Dietary levels of all-trans retinol affect retinoid nuclear receptor expression and skeletal development in European sea bass larvae. British Journal of Nutrition, 2005; 93(06), 791 10.1079/BJN20051421 16022748

[16] Villeneuve L, Gisbert E, Moriceau J, Cahu CL, Infante JL. Intake of high levels of vitamin A and polyunsaturated fatty acids during different developmental periods modifies the expression of morphogenesis genes in European sea bass (Dicentrarchus labrax). British Journal of Nutrition, 2006; 95(04), 677 10.1079/BJN20051668 16571146

[17] Pittenger MF, Mackay AM, Beck SC, Jaiswal RK, Douglas R, Mosca J D, et al Multilineage potential of adult human mesenchymal stem cells. Science (New York, N.Y.), 1999; 284(5411), 143–147. 10.1126/science.284.5411.143 10102814

[18] Ytteborg E, Vegusdal A, Witten PE, Berge GM, Takle H, Østbye TK, et al Atlantic salmon (Salmo salar) muscle precursor cells differentiate into osteoblasts in vitro: Polyunsaturated fatty acids and hyperthermia influence gene expression and differentiation. Biochimica et Biophysica Acta (BBA)—Molecular and Cell Biology of Lipids, 2010; 1801(2), 127–137. 10.1016/j.bbalip.2009.10.001bmed/10102814 19833228

[19] Matsubara T, Kida K, Yamaguchi A, Hata K, Ichida F, Meguro H, et al BMP2 regulates osterix through msx2 and runx2 during osteoblast differentiation. Journal of Biological Chemistry, 2008; 283(43), 29119–29125. 10.1074/jbc.M801774200 18703512 PMC2662012

[20] Chen L, Jacquet R, Lowder E, Landis WJ. Refinement of collagen–mineral interaction: A possible role for osteocalcin in apatite crystal nucleation, growth and development. Bone, 2015; 71, 7–16. 10.1016/j.bone.2014.09.021 25284158

[21] Fonseca VG, Laizé V, Valente MS, Cancela ML. Identification of an osteopontin-like protein in fish associated with mineral formation. FEBS Journal, 2007; 274(17), 4428–4439. 10.1111/j.1742-4658.2007.05972.x 17680811

[22] Vieira FA, Thorne MAS, Stueber K, Darias M, Reinhardt R, Clark MS. Comparative analysis of a teleost skeleton transcriptome provides insight into its regulation. General and Comparative Endocrinology, 2013; 191, 45–58. 10.1016/j.ygcen.2013.05.025 23770218

[23] Berendsen AD, Olsen BR. How vascular endothelial growth factor-A (VEGF) regulates differentiation of mesenchymal stem cells. Journal of Histochemistry and Cytochemistry, 2014; 62(2), 103–108. 10.1369/0022155413516347 24309509 PMC3902099

[24] Chen Q, Shou P, Zheng C, Jiang M, Cao G, Yang Q, et al Fate decision of mesenchymal stem cells: adipocytes or osteoblasts? Cell Death and Differentiation, 2016; 23(7), 1128–1139. 10.1038/cdd.2015.168 26868907 PMC4946886

[25] Grygiel-Górniak B. Peroxisome proliferator-activated receptors and their ligands: nutritional and clinical implications—a review. Nutrition Journal, 2014; 13(1), 17 10.1186/1475-2891-13-17 24524207 PMC3943808

[26] Otto TC, Lane MD. Adipose Development: From Stem Cell to Adipocyte. Critical Reviews in Biochemistry and Molecular Biology, 2005; 40(4), 229–242. 10.1080/10409230591008189 16126487

[27] Sánchez-Gurmaches J, Cruz-Garcia L, Gutiérrez J, Navarro I. mRNA expression of fatty acid transporters in rainbow trout: in vivo and in vitro regulation by insulin, fasting and inflammation and infection mediators. Comparative Biochemistry and Physiology Part A: Molecular and Integrative Physiology, 2012; 163(2), 177–188. 10.1016/j.cbpa.2012.06.010 22771331

[28] Gregoire FM, Smas CM, Sul HS. Understanding adipocyte differentiation. Physiological Reviews, 1998; 78(3), 783–809. 10.1152/physrev.1998.78.3.783 9674695

[29] Gustafson B, Gogg S, Hedjazifar S, Jenndahl L, Hammarstedt A, Smith U. Inflammation and impaired adipogenesis in hypertrophic obesity in man. American Journal of Physiology-Endocrinology and Metabolism, 2009; 297(5), E999–E1003. 10.1152/ajpendo.00377.2009 19622783

[30] Spiegelman BM, Flier JS. Adipogenesis and obesity: rounding out the big picture. Cell, 1996; 87(3), 377–389. 8898192 10.1016/s0092-8674(00)81359-8

[31] Kelly O, Cusack S, Jewell C, Cashman KD. The effect of polyunsaturated fatty acids, including conjugated linoleic acid, on calcium absorption and bone metabolism and composition in young growing rats. British Journal of Nutrition, 2003; 90(04), 743 10.1079/BJN2003951 13129442

[32] Li Y, Watkins BA. Conjugated linoleic acids alter bone fatty acid composition and reduce ex vivo prostaglandin E2 biosynthesis in rats fed n-6 or n-3 fatty acids. Lipids, 1998; 33(4), 417–425. 10.1007/s11745-998-0223-9 9590630

[33] Watkins B, Lippman H, Le Bouteiller L, Li Y, Seifert M. Bioactive fatty acids: role in bone biology and bone cell function. Progress in Lipid Research, 2001; 40(1–2), 125–148. 10.1016/S0163-7827(00)00016-3 11137570

[34] Watkins BA, Li Y, Lippman HE, Feng S. Modulatory effect of omega-3 polyunsaturated fatty acids on osteoblast function and bone metabolism. Prostaglandins, Leukotrienes and Essential Fatty Acids, 2003; 68(6), 387–398. 10.1016/S0952-3278(03)00063-2 12798659

[35] Capilla E, Teles-García Á, Acerete L, Navarro I, Gutiérrez J. Insulin and IGF-I effects on the proliferation of an osteoblast primary culture from sea bream (Sparus aurata). General and Comparative Endocrinology, 2011; 172(1), 107–114. 10.1016/j.ygcen.2011.03.020 21447336

[36] Riera-Heredia N, Martins R, Mateus AP, Costa RA, Gisbert E, Navarro I, et al Temperature responsiveness of gilthead sea bream bone an in vitro and in vivo approach. Scientific Reports, 2018; 8(1), 11211 10.1038/s41598-018-29570-9 30046119 PMC6060158

[37] Salmerón C, Acerete L, Gutiérrez J, Navarro I, Capilla E. Characterization and endocrine regulation of proliferation and differentiation of primary cultured preadipocytes from gilthead sea bream (Sparus aurata). Domestic Animal Endocrinology, 2013; 45(1), 1–10. 10.1016/j.domaniend.2013.02.002 23535263

[38] Salmerón C, Riera-Heredia N, Gutiérrez J, Navarro I, Capilla E. Adipogenic gene expression in gilthead sea bream mesenchymal stem cells from different origin. Frontiers in Endocrinology, 2016; 7, 113 10.3389/fendo.2016.00113 27597840 PMC4992700

[39] Ytteborg E, Todorcevic M, Krasnov A, Takle H, Kristiansen IO, Ruyter B. Precursor cells from Atlantic salmon (Salmo salar) visceral fat holds the plasticity to differentiate into the osteogenic lineage. Biology Open, 2015; 4(7), 783–791. 10.1242/bio.201411338 25948755 PMC4571100

[40] Brust R, Shang J, Fuhrmann J, Bass J, Cano A, Heidari Z, et al. A structural mechanism for directing inverse agonism of PPARγ. Preprint. Available from bioRxiv, 10.1101/245852 Cited 17 December 2018. PMC6224492 30409975

[41] Lee G, Elwood F, McNally J, Weiszmann J, Lindstrom M, Amaral K, et al T0070907, a selective ligand for peroxisome proliferator-activated receptor γ, functions as an antagonist of biochemical and cellular activities. Journal of Biological Chemistry, 2002; 277(22), 19649–19657. 10.1074/jbc.M200743200 11877444

[42] Ouadah-Boussouf N, Babin PJ. Pharmacological evaluation of the mechanisms involved in increased adiposity in zebrafish triggered by the environmental contaminant tributyltin. Toxicology and Applied Pharmacology, 2016; 294, 32–42. 10.1016/j.taap.2016.01.014 26812627

[43] Pfaffl MW. A new mathematical model for relative quantification in real-time RT-PCR. Nucleic Acids Research, 2001; 29(9), 45e–45. 10.1093/nar/29.9.e45 PMC55695 11328886

[44] Casado-Díaz A, Santiago-Mora R, Dorado G, Quesada-Gómez JM. The omega-6 arachidonic fatty acid, but not the omega-3 fatty acids, inhibits osteoblastogenesis and induces adipogenesis of human mesenchymal stem cells: potential implication in osteoporosis. Osteoporosis International, 2013; 24(5), 1647–1661. 10.1007/s00198-012-2138-z 23104199

[45] Viegas MN, Dias J, Cancela ML, Laize V. Polyunsaturated fatty acids regulate cell proliferation, extracellular matrix mineralization and gene expression in a gilthead seabream skeletal cell line. J. Appl. Ichthyol., 2012; 28 pp. 427–432

[46] Bouraoui L, Gutiérrez J, Navarro I. Regulation of proliferation and differentiation of adipocyte precursor cells in rainbow trout (Oncorhynchus mykiss). Journal of Endocrinology, 2008; 198(3), 459–469. 10.1677/JOE-08-0264 18579724

[47] Oku H, Tokuda M, Okumura T, Umino T. Effects of insulin, triiodothyronine and fat soluble vitamins on adipocyte differentiation and LPL gene expression in the stromal-vascular cells of red sea bream, Pagrus major. Comparative Biochemistry and Physiology Part B: Biochemistry and Molecular Biology, 2006; 144(3), 326–333. 10.1016/j.cbpb.2006.03.008 16716627

[48] Vegusdal A, Sundvold H, Gjøen T, Ruyter B. An in vitro method for studying the proliferation and differentiation of Atlantic salmon preadipocytes. Lipids, 2003; 38(3), 289–296. 12784870 10.1007/s11745-003-1063-3

[49] Matsubara Y, Sato K, Ishii H, Akiba Y. Changes in mRNA expression of regulatory factors involved in adipocyte differentiation during fatty acid induced adipogenesis in chicken. Comparative Biochemistry and Physiology Part A: Molecular & Integrative Physiology, 2005; 141(1), 108–115. 10.1016/J.CBPB.2005.04.013 15922639

[50] Todorcević M, Vegusdal A, Gjøen T, Sundvold H, Torstensen BE, Kjaer MA, et al Changes in fatty acids metabolism during differentiation of Atlantic salmon preadipocytes; effects of n-3 and n-9 fatty acids. Biochim Biophys Acta, 2008; 1781:326–35. 10.1016/j.bbalip.2008.04.014 18503782

[51] Kim HK, Della-Fera M, Lin J, Baile CA. Docosahexaenoic acid inhibits adipocyte differentiation and induces apoptosis in 3T3-L1 preadipocytes. The Journal of Nutrition, 2006; 136(12), 2965–2969. 10.1093/jn/136.12.2965 17116704

[52] Jeon MJ, Kim JA, Kwon SH, Kim SW, Park KS, Park SW, et al Activation of peroxisome proliferator-activated receptor-gamma inhibits the Runx2-mediated transcription of osteocalcin in osteoblasts. The Journal of Biological Chemistry, 2003; 278(26), 23270–23277. 10.1074/jbc.M211610200 12704187

[53] Lecka-Czernik B, Gubrij I, Moerman EJ, Kajkenova O, Lipschitz DA, Manolagas SC, et al Inhibition of Osf2/Cbfa1 expression and terminal osteoblast differentiation by PPARgamma2. Journal of Cellular Biochemistry, 1999; 74(3), 357–371. 10412038

[54] Zhang X, Yang M, Lin L, Chen P, Ma KT, Zhou CY, et al Runx2 overexpression enhances osteoblastic differentiation and mineralization in adipose-derived stem cells in vitro and in vivo. Calcified Tissue International, 2006; 79(3), 169–178. 10.1007/s00223-006-0083-6 16969589

[55] Schmitz G, Ecker J. The opposing effects of n−3 and n−6 fatty acids. Progress in Lipid Research, 2008; 47(2), 147–155. 10.1016/j.plipres.2007.12.004 18198131

[56] Lefterova MI, Lazar MA. New developments in adipogenesis. Trends in Endocrinology and Metabolism, 2009; 20(3), 107–114. 10.1016/j.tem.2008.11.005 19269847

[57] Moreno-Navarrete JM, Fernández-Real JM. Adipocyte differentiation. Adipose Tissue Biology, 2012; 17–38 10.1007/978-1-4614-0965-6_2

[58] Lenhard JM. PPAR gamma/RXR as a molecular target for diabetes. Receptors Channels, 2001; 7, 249–258. 11697231 10.1111/j.1651-2227.2001.tb03250.x

[59] Choi IH, Chung CY, Cho TJ, Yoo WJ. Angiogenesis and mineralization during distraction osteogenesis. Journal of Korean Medical Sciences, 2002; 17, 435447 10.3346/jkms.2002.17.4.435 12172035 PMC3054899

[60] Nuttall ME, Gimble JM. Controlling the balance between osteoblastogenesis and adipogenesis and the consequent therapeutic implications. Current Opinion in Pharmacology, 2004; 4, 290–294. 10.1016/j.coph.2004.03.002 15140422

[61] Gil Martens L, Lock EJ, Fjelldal PG, Wargelius A, Araujo P, Torstensen BE, et al Dietary fatty acids and inflammation in the vertebral column of Atlantic salmon, Salmo salar L., smolts: a possible link to spinal deformities. Journal of Fish Diseases, 2010; 33(12), 957–972. 10.1111/j.1365-2761.2010.01201.x 21091723

[62] Ytteborg E, Baeverfjord G, Torgersen J, Hjelde K, Takle H. Molecular pathology of vertebral deformities in hyperthermic Atlantic salmon (Salmo salar). BMC Physiology, 2010; 10, 12 10.1186/1472-6793-10-12 20604915 PMC2914708

[63] Ytteborg E, Torgersen J, Baeverfjord G, Takle H. Morphological and molecular characterization of developing vertebral fusions using a teleost model. BMC Physiology, 2010; 10, 13 10.1186/1472-6793-10-13 20604916 PMC2909226

[64] Ytteborg E, Torgersen JS, Pedersen ME, Baeverfjord G, Hannesson KO, Takle H. Remodeling of the notochord during development of vertebral fusions in Atlantic salmon (Salmo salar). Cell and Tissue Research, 2010; 342,363–376. 10.1007/s00441-010-1069-2 21086140

[65] Helland S, Denstadli V, Witten PE, Hjelde K, Storebakken T, Skrede A, et al Hyper dense vertebrae and mineral content in Atlantic salmon (Salmo salar L.) fed diets with graded levels of phytic acid. Aquaculture, 2006; 261, 603–614. 10.1016/j.aquaculture.2006.08.027

[66] Bou M, Montfort J, Le Cam A, Rallière C, Lebret V, Gabillard JC, et al Gene expression profile during proliferation and differentiation of rainbow trout adipocyte precursor cells. BMC Genomics, 2017; 18(1), 347 10.1186/s12864-017-3728-0 28472935 PMC5418865

[67] Lim HJ, Lee S, Lee KS, Park JH, Jang Y, Lee EJ, et al PPARγ activation induces CD36 expression and stimulates foam cell like changes in rVSMCs. Prostaglandins and Other Lipid Mediators, 2006; 80(3–4), 165–174. 10.1016/j.prostaglandins.2006.06.006 16939881

[68] Huang TS, Todorčević M, Ruyter B, Torstensen BE. Altered expression of CCAAT/enhancer binding protein and FABP11 genes during adipogenesis in vitro in Atlantic salmon (Salmo salar). Aquaculture Nutrition, 2010; 16(1), 72–80. 10.1111/j.1365-2095.2008.00642.x

[69] Lelliott C, Vidal-Puig AJ. Lipotoxicity, an imbalance between lipogenesis de novo and fatty acid oxidation. International Journal of Obesity and Related Metabolic Disorders: Journal of the International Association for the Study of Obesity, 2004; 28 Suppl 4(S4), S22–8. 10.1038/sj.ijo.0802854 15592482

[70] Her GM, Pai WY, Lai CY, Hsieh YW, Pang HW. Ubiquitous transcription factor YY1 promotes zebrafish liver steatosis and lipotoxicity by inhibiting CHOP-10 expression. Biochimica et Biophysica Acta (BBA)—Molecular and Cell Biology of Lipids. 2013; 1831:1037–1051. 10.1016/j.bbalip.2013.02.002 23416188

[71] Babaahmadi Rezaei H, Doosti M, Aminian M, Shabani P. Compare the effect of eicosapentaenoic acid and oxidized low-density lipoprotein on the expression of CD36 and peroxisome proliferator-activated receptor gamma. Iranian Biomedical Journal, 2013; 17(2), 84–92. 10.6091/ibj.11322.2013 23567850 PMC3677680

[72] Kuda O, Rossmeisl M, Kopecky J. Omega-3 fatty acids and adipose tissue biology. Molecular Aspects of Medicine, 2018; 64, 147–160. 10.1016/j.mam.2018.01.004 29329795

[73] Okuno A, Tamemoto H, Tobe K, Ueki K, Mori Y, Iwamoto K, et al Troglitazone increases the number of small adipocytes without the change of white adipose tissue mass in obese Zucker rats. Journal of Clinical Investigation, 1998; 101(6), 1354–1361. 10.1172/JCI1235 9502777 PMC508690

[74] De Souza CJ, Eckhardt M, Gagen K, Dong M, Chen W, Laurent D, et al Effects of pioglitazone on adipose tissue remodeling within the setting of obesity and insulin resistance. Diabetes, 2001; 50(8), 1863–1871. 10.2337/diabetes.50.8.1863 11473050

[75] Gimble JM, Nuttall ME. The relationship between adipose tissue and bone metabolism. Clinical Biochemistry, 2012; 45, 874–879. 10.1016/j.clinbiochem.2012.03.006 22429519

[76] Moerman EJ, Teng K, Lipschitz DA, Lecka-Czernik B. Aging activates adipogenic and suppresses osteogenic programs in mesenchymal marrow stroma/stem cells: the role of PPAR-gamma2 transcription factor and TGF-beta/BMP signaling pathways. Aging Cell, 2004; 3, 379–389. 10.1111/j.1474-9728.2004.00127.x 15569355 PMC1850101

