# Supplementary figures and images for: Fatty acids from fish or vegetable oils promote the adipogenic fate of mesenchymal stem cells derived from gilthead sea bream bone potentially through different pathways
Source: PLoS One. 2019 Apr 24;14(4):e0215926. doi: 10.1371/journal.pone.0215926 (PMC6481918; doi:10.1371/journal.pone.0215926)

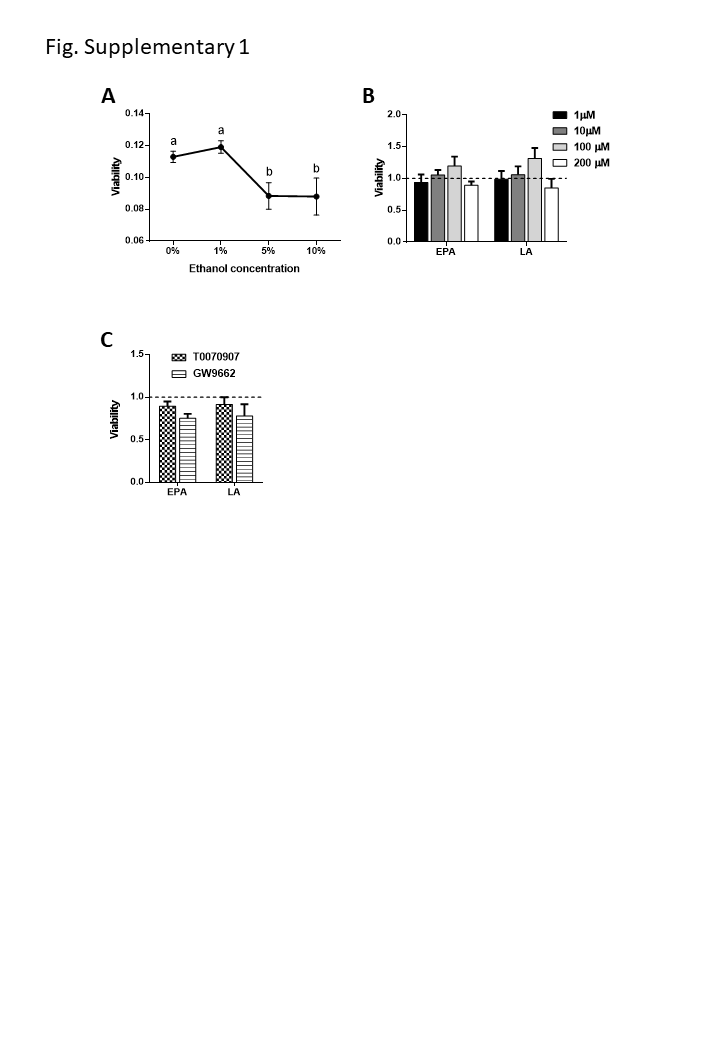

Supplement: S1 Fig — Viability of gilthead sea bream bone-derived cells at day 4 determined by means of the MTT assay. Cells were treated (A) for 24 h with different concentrations of ethanol; or for 6 h (B) with different concentrations of selected fatty acids (EPA and LA) or were left untreated as control (dashed line), and (C) with the fatty acids EPA or LA in the absence (dashed line) or the presence of a PPARγ antagonist (T0070907 or GW9662). Data are shown as mean + SEM (n = 3). Significant differences (p<0.05) among concentrations are indicated by different letters. Asterisks indicate significant differences (p<0.05) with the corresponding control. EPA: eicosapentaenoic acid; LA: linoleic acid. (TIF) [file pone.0215926.s001.tif]
